# A look at food security in China

**DOI:** 10.1038/s41538-018-0012-x

**Published:** 2018-02-20

**Authors:** Kai Cui, Sharon P. Shoemaker

**Affiliations:** 1 California Institute of Food and Agricultural Research, University of California Davis, Davis, CA USA; 2 Antai College of Economics and Management, Shanghai Jiao Tong University, Shanghai Shi, China

**Keywords:** Agriculture, Environmental sciences

Who will feed China? This important question was raised by Dr. Lester R. Brown in his famous book by the same title.^1^ It is still a valid question today as food security continues to be one of the highest priorities for this most populated country in the world. Food production (namely, grain), inputs (fertilizer, water) and outputs (state of environment) are vital elements to a sustainable food system that must be considered along with consumers (people, animals) and where consumers are located (urban, rural). In the past century, China experienced numerous food shortages, then corrected them by implementing a quota system (1955–1993) and land contract reform (1981) that incentivized farmers. Total grain output increased 74% from 354 million tons in 1982 to 618 million tons in 2017, surpassing the growth of its population by about 34%.^2,3^

Today, China feeds 20% of the world’s population on 7% of the world’s farmlands.^2,3^ To accomplish this feat, China paid a heavy price. China’s excessive and inefficient use of chemical fertilizers, increasing 3-fold in the past three decades, efficiencies averaging at 32% compared to world average of 55%,^2^ contributed to its current harmful state of environmental pollution.

Similarly, China’s water situation is problematic with low efficiency, poor quality and unequal distribution throughout the country. China’s available water supply per person is only 2050 m^3^ or 25% of the world’s per capita average. Irrigation of rural crops accounts for 60% of China’s total water demand with inefficient delivery of the order of 30–40%, compared to 70–80% for developed countries. In some regions of northern China, where water is scarce, excessive amounts of groundwater are being directed to agriculture. Therefore, it is urgent that China proactively consider how to achieve food security through a balance of resource management, environmental protection and sustainable agricultural development.

In the future, improvement in grain productivity will depend more on technology adaptation than increasing resource inputs. China is making positive improvements to the quality of its soil, and to reducing its water and fertilizer use on crops. An accelerating rate of land transfers is leading to the construction of huge, modern farms with large scale planting and mechanization should further improve agricultural production efficiency. Advances in digital technologies can enable precision agricultural practices to increase grain production on the order of 10%. Moreover, if genetically modified crops (GM-corn and GM-soybean) are permitted to grow in China, then they could ensure adequate grain production and supply with more efficient use of natural resources, while at the same time, moderating the use levels of agricultural chemicals, thus, reducing environmental pollution.

Among the three major cereal crops grown in China, the self-sufficiency ratio of wheat, rice and corn is about 95%. In contrast, about 80% of consumed soybean and other agri-products, such as milk and sugar, are imported to China. It is interesting that soybean imports significantly increased from 0.3 million tons in 1995 to 95 million tons in 2017, presently accounting for two-thirds of the world’s soybean market. The per unit area yield of soybean is much less than that of other major crops, about 1/3 of wheat, 1/4 of rice and 1/5 of corn. With China’s shift to importing rather than growing soybean, a total of 50 million hectares of fertile cropland (40% of China’s total arable land^−^120 million hectares^1^) freed up for growing other higher yielding crops.

A critical question is “How much grain will Chinese consumers require in the future?” The answer depends on China’s population growth and its concomitant food demands. In 1980 when the government established the “one child policy”; it based population growth on models that predicted the birthrate would fall (from above 1.8 before 2000) and predicted a population of 1.6 billion in China by 2050.^4,5^ It now seems that this prediction was greatly flawed and the total birthrate has been no more than 1.4. Thus, China’s population was only 1.37 billion at the end of 2016. A peak in China’s population growth is now expected by 2025 with a total population of about 1.42 billion and then India is expected to replace China as the world’s most populated country.^6^ After 2025, China’s population is expected to decline rapidly and may fall to 1.2 billion by 2050, and even further to 0.6 billion by 2100.^7^

Severe problems due to the evolving Chinese aging population and low birth rate may soon arise, even with the “one child policy” being repealed in 2015. Families can now have a second child but many choose not to, because they cannot afford the high cost of raising children. Thus, some demographers now predict that the second child policy may lead to only 2-4 million additional people in China each year for the next 10 years.

China’s accelerated increase in urban population, relative to that in the rural countryside, is and will continue have an impact on food consumption. China’s urbanization rate reached 57% in 2016, and may increase to 65% by 2025 and 80% by 2050. Who will produce the food in the countryside given these statistics? Even though there is a leveling off in China’s population, with an ever-aging population and a lower demand for food by city dwellers compared to people in the countryside, there still needs to be an educated workforce devoted to producing, processing, packaging and distributing the food safely and affordably.

With China’s population approaching its peak, grain consumption is expected to decrease starting this year. According to consumption patterns of grain in China, direct grain consumption accounts for only 30%, the remainder of which is found in processed grain products for food and feed. Thus, the general consumption of food products has been stable for the past 3 years. In contrast, the total production of meat, milk, and beer has gone down to 4, 6 and 7%, respectively from 2014 to 2016.^8^ This suggests that total food consumption in China has reached or is approaching a maximum. These results can be explained by the fact that the Chinese people primarily consume a vegetarian diet of 400 kg of food consumed per capita. Based on the annual grain output in China, food consumption per capita reached 350 kg in 2004, 400 kg in 2010 and 450 kg in 2015. Believe it or not, China now has the highest number of obese people in the world. In the coming decade, annual grain production should stabilize at about 600 million tons which will allow food consumption per capita above 430 kg.

Finally, China is wasting food. About one-sixth of the total grain produced in China is wasted annually in the production, processing and transportation of food because of poor equipment and logistical issues. The amount of the “wasted grain” is equivalent to about one-sixth of the total grain produced in China. In addition, there is also considerable waste in the form of consumer left-overs and waste from outdated food removed from supermarkets. Although there is no reliable data on food waste yet available in China to analyze this trend, it is anticipated that future improvements to China’s industrialization and consumer behavior, will result in a reduction of food waste.

In summary, China’s food and agricultural system is undergoing a historic transformation and will continue to do so into the foreseeable future. In recent years, there has been no space available in some state-owned grain reserves and some difficulty in selling grains. The price of corn in 2017 fell to about 35% of its price in 2015. China plans to reduce 3.3 million hectares of farmland for planting maize (3% of countrywide farmland) from 2016 to 2020 reducing environmental pollution. This action also reflects China’s government new confidence in its national food security. Newly accepted imports, such as rice from USA, will relieve some pressure for domestic production. China has successfully defended and overcome the shocks of floods with “grain” being its “levee system” and, although the water level is expected to remain high over the next decade, it is expected to decline thereafter. Thus, we are cautiously optimistic about the future of China’s food security, but China must place close attention to the coordinated sustainable development of its people, resources and environment.
